# Stroke mimics: incidence, aetiology, clinical features and treatment

**DOI:** 10.1080/07853890.2021.1890205

**Published:** 2021-03-06

**Authors:** Brian H. Buck, Naveed Akhtar, Anas Alrohimi, Khurshid Khan, Ashfaq Shuaib

**Affiliations:** a Department of Medicine (Neurology), University of Alberta, Edmonton, Canada; b Neurological Institute, Hamad Medical Corporation, Doha, Qatar; c Department of Medicine (Neurology), King Saud University, Riyadh, Saudi Arabia

**Keywords:** Stroke, mimics, MRI, recurrence, TIA

## Abstract

Mimics account for almost half of hospital admissions for suspected stroke. Stroke mimics may present as a functional (conversion) disorder or may be part of the symptomatology of a neurological or medical disorder. While many underlying conditions can be recognized rapidly by careful assessment, a significant proportion of patients unfortunately still receive thrombolysis and admission to a high-intensity stroke unit with inherent risks and unnecessary costs. Accurate diagnosis is important as recurrent presentations may be common in many disorders. A non-contrast CT is not sufficient to make a diagnosis of acute stroke as the test may be normal very early following an acute stroke. Multi-modal CT or magnetic resonance imaging (MRI) may be helpful to confirm an acute ischaemic stroke and are necessary if stroke mimics are suspected. Treatment in neurological and medical mimics results in prompt resolution of the symptoms. Treatment of functional disorders can be challenging and is often incomplete and requires early psychiatric intervention.

## The significance of the clinical problem

Stroke is one of the most common diseases affecting one in four people during their lifetime [1]. Most strokes are due to reduction or interruption of blood flow to the brain (ischaemic stroke). A small minority may result from thrombosis in medium or large cerebral veins. Approximately, 20–30% of strokes are haemorrhages and results from damage to small or medium-size vessels [1]. Stroke is a medical emergency and presents with focal neurological deficits. Immediate evaluation, confirmation of diagnosis and treatment to re-establish blood flow leads to improvement in symptoms and prevention of brain damage [2]. The diagnosis of acute ischaemic stroke is however not always straightforward. Similar symptoms may develop in a number of medical conditions commonly referred to as “stroke mimics” (see Table 1). It is essential to entertain stroke mimics in the differential diagnosis when treating an acute suspected stroke to avoid the inappropriate use of expensive and potentially harmful medications. This becomes particularly important with telestroke and in hospitals with limited acute stroke experience [3].

**Table 1.** Conditions that may be confused as acute stroke (stroke mimics).

Conditions	Types	Examples	
	Recrudescence		
	Brain disorders without structural lesions	Migraine	Peripheral vertigo
		Focal neuropathies	Transient global amnesia
		Bell’s palsy	
	Brain disorders with structural lesions	Posterior reversible Vasoconstrictive syndrome (PRES)	Multiple sclerosis and other demyelinating diseases
		Seizure disorder/TPMA^a^ (Figure 4)	Arterio-venous malformation
		Eye disorders	Meningeal disease
		Sub-acute/acute bacterial endocarditis	Atrial myxoma
		Channelopathies	Trauma
		Epidural/subdural haemorrhage (Figure 3)	Aortic dissection
Medical mimics		Hypertensive crisis	Arnold-Chiari malformation
		Brain neoplasm	Stroke-like migraine attacks after radiation therapy (SMART) syndrome
	Systemic medical conditions	Electrolytes dysfunction	Alcohol
		Metabolic/toxic disorders	Acute liver failure
		Hypoglycaemia and hyperglycaemia	
	Negative investigations (stroke misdiagnosis)		
Functional mimics		Depression	Somatization
		Stress	Psychiatric complications of neurological conditions
		Anxiety disorder	
		Chronic pain/fatigue	

^a^TPMA: transient peri-ictal MRI abnormalities.

The frequency of stroke mimics is variable and depends where the diagnosis is made and can for 20–50% of cases of acute suspected stroke depending if the patients are evaluated by the emergency personal or stroke physicians [3–5]. Stroke mimics can broadly be classified into two categories. Medical mimics are more common and comprise 50–80% of cases in most large series [4,6]. Functional mimics or conversion disorders are less frequent [7–10]. Although they have characteristic clinical features can sometimes be very difficult to differentiate from an ischaemic stroke (see Table 2). The separation of “new focal neurological symptoms” in the presence of an old stroke, also known as recrudescence, can be particularly challenging. This frequently develops in the settings of an acute infection or metabolic dysfunction and can occur weeks to years following a stroke [11]. Diagnosis may sometimes require magnetic resonance imaging (MRI) to determine if a new stroke is responsible for the focal symptoms. This is important when thrombolysis is being contemplated.

**Table 2.** Clinical characteristics differentiating stroke from mimic.

Characteristic	Stroke	Mimics
Age and sex	Older age (male = female)	Younger age (females > males)
Level of consciousness	Awake	Altered level of consciousness
Onset and progress	Acute and sudden	Gradual in onset
Symptoms severity	Severe at onset	Fluctuations in severity are common
Risk factors	Vascular risk factors	Migraine, seizure, systemic illness
Vascular territory	Vascular syndromes	No vascular distribution
Blood pressures at presentation	Increase blood pressure at onset is common	Blood pressure usually not increased
Signs and symptoms	Weakness (pyramidal distribution), aphasia and visual filed defects	Sensory, vertigo (dizziness) and visual
Involuntary movements	Uncommon	May have involuntary movements
Imaging	Imaging shows ischaemic lesions	Imaging helpful in diagnosis
EEG	EEG may show slowing over the affected area	May show spike and wave in seizures Unilateral facial twitching and lip-smacking Giveaway weakness Arm drift/abrupt fall without pronation

Stroke mimics have less clearly defined neurological symptoms that typically do not adhere to well-defined stroke syndromes [12]. The suddenness at onset is not always evident, fluctuations in severity are common and systemic signs including drowsiness, confusion, agitation and fever may be present [6]. Common presenting symptoms include vertigo and dizziness, altered level of consciousness, paraesthesia and numbness, monoplegia, speech dysfunction, limb ataxia, headache and visual disturbances (see Table 2). There is often a previous history of seizures, migraine, depression or other psychiatric disorders or dementia [4]. Mimics can be particularly difficult to differentiate from acute stroke when symptoms are brief and resolve before the patient is examined, especially when advanced brain imaging including MRI is normal. Prompt identification that symptoms are secondary to a stroke mimic and appropriate treatment of the underlying condition will lead to avoidance of potential misdiagnosis and the unnecessary long-term use of antithrombotic and other stroke prevention medication.

## Assessment of the patient with suspected acute stroke

**Figure 1.**
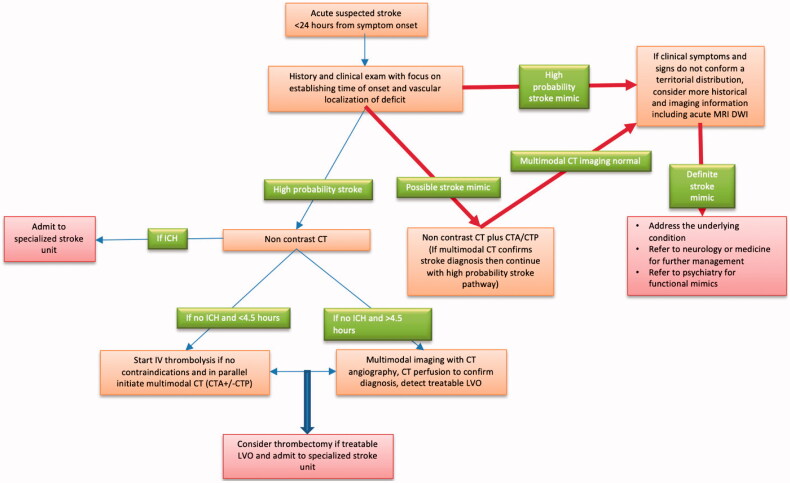
Suggested approach to evaluation of acute stroke patient where mimic is suspected.

Acute stroke requires immediate diagnosis, especially in patients arriving within the time window for reperfusion treatment. This may be up to 9 h (or wake-up) for thrombolysis [13] and 24 h for thrombectomy in selected cases with large vessel intracranial occlusion [1]. The emergent evaluation of patients with suspected stroke involves the determination of the time of onset and the rapidity with which the symptoms developed [1]. The initial examination confirms the severity of symptoms and the anatomical localization (cortical *vs.* sub-cortical) and the vascular distribution (anterior circulation *vs.* posterior circulation). The first brain imaging for most patients is a non-contrast computerized tomography (CT) scan which is completed within minutes of the arrival of the patient to the emergency department (ED). Very early, the scan may show no evidence of an acute stroke [5,14]. Further CT sequences including CT-angiography (CTA) and CT-perfusion (CTP) [15] can make the diagnosis especially in the presence of an intracranial large artery occlusion and perfusion abnormalities [5,16]. Diffusion-weighted MRI (DWI-MRI) is the most sensitive diagnostic tool and shows ischaemic changes very early but is not widely available when required urgently [5]. Abnormalities detected on MRI can be very useful in diagnosis, especially when symptoms are transient, and can be very useful in determining whether the aetiology was ischaemic. The presence of MRI-defined ischaemic lesions in addition to confirmation of diagnosis is associated with a significantly higher risk of future stroke risk [17]. MRI is however not necessary in the assessment of most stroke mimic patients as the clinical examination and the CT imaging is sufficient to make a firm diagnosis. Figure 1 shows a schematic overview of imaging in acute stroke.

The positive effects of thrombolysis in acute stroke are time-dependent and the treatment must be initiated quickly once the CT is completed. There is therefore increased risk that stroke mimics may also be inadvertently thrombolysed. Early recognition of stroke mimics is therefore important to avoid the risk of intracranial haemorrhage (ICH) and the cost associated with treatment with tissue plasminogen activator (tPA) and the additional cost of care in the Intensive Care Unit or a Stroke Unit. The use of multimodal brain imaging, including CTA and CTP may improve the detection of stroke mimics in such situations [18–20**].**

The main goal of pre-hospital stroke systems is to ensure that treatable patients with acute suspected stroke are transported to primary and comprehensive stroke centres with rapid access to diagnostic imaging and reperfusion therapies. To accomplish this goal prehospital dispatch and EMS stroke algorithms are designed to maximize sensitivity and minimize the numbers of treatable acute strokes not directly transported to stroke centres. The intentional prehospital overtriage of patients with acute stroke presentations to EDs at primary and comprehensive stroke centres does however come at the cost of diverting patients that ultimately will be diagnosed by a non-stroke condition to stroke centres. As a result, the proportion of stroke mimics depends on the setting where patients with stroke presentations are evaluated. It is the highest when the emergency dispatch is activated by suspected stroke patients or their attendants. The rates remain high with assessment by paramedics in the ambulance as the patient is transported to the hospital as well. Even in the ED, more than 40% of patients may be diagnosed with non-stroke conditions after completion of the initial investigations and transient ischaemic attacks, ischaemic stroke or haemorrhage have been excluded [21].

The four important time-critical situations where a distinction between an acute stroke and a stroke-mimic is important and includes the dispatch call by patient/attendant, the initial assessment by the ambulance paramedics, ED evaluation and remote telestroke assessment. These are discussed in more detail separately below.

### Pre-hospital dispatch evaluation of stroke mimics

Emergency medical dispatch services (MDS) is very often the first point of contact for patients with suspected stroke or their attendant and plays an important role in the optimization of delivery of care. Frequently the caller uses vague, non-specific or potentially distractor words making it difficult for the dispatcher to recognize the diagnosis [22]. The dispatchers are very exposed to a wide variety of incoming calls and have a difficult task to rapidly identify stroke symptoms and prioritize ambulance dispatch. Interestingly, when the dispatcher suspects that the symptoms may suggest a stroke, the patient is more likely to receive expedited care [23]. In a systemic review of 25 studies, stroke recognition by medical dispatchers was modest with a sensitivity of 41–83% and specificity of 42–68% [24]. These may be overestimated as a more recent comprehensive study showed a specificity of only 21% for correct diagnosis, following ambulance activation by MDS [25]. This low degree of accuracy is understandable as the MDS have personal with limited medical expertise and may not differentiate acute stroke from a number of acute emergencies with neurological symptoms.

The medical dispatcher has the difficult task of making diagnosis decisions to activate the ambulance service. An acute stroke is one of several important emergencies that require immediate attention. It may not always be the first condition in their differential diagnosis when presented with focal neurological symptoms. The dispatcher may consider medical conditions that mimic a stroke to be as important and may fail to appreciate the distinction between an acute stroke requiring thrombectomy or status epilepticus requiring immediate attention when activating the ambulance. The real danger is the failure to recognize an acute stroke and thus the delay in fast transfer to the appropriate stroke-ready hospital. This may occur in 50% of patients that are admitted to hospitals with an acute stroke [26]. The positive predictive value of identifying an acute stroke is also, unfortunately, lower than 50%. The remainder of patients being stroke mimics [26,27].

Most MDS facilities have computerized algorithms that facilitate the diagnosis based on the presenting symptoms of patients. The automated system for stroke, the Medical Priority Dispatch System (MPDS, Priority Dispatch Incorporated) was implemented in 2000 and is used throughout the USA and Canada. There appears to be very minimal information for recognition of stroke symptoms in the MDPS. Surprisingly, the frequency of stroke mimics is higher when MDS is activated by residents from nursing homes, a group of subjects in whom the risk of an acute stroke is high. In a recent study of 419 nursing home patients that were admitted to ED with suspected stroke, 66% were stroke mimics. Infections (24%) and seizures (20%) were the most frequent aetiologies [24].

We have recent experience with MDS activation of our mobile stroke ambulance (MSU). The MSU is activated when patients, a family member or bystander calls the MDS for a suspected stroke. In the initial three years of experience with our MSU, 90% were false activations. In 80%, paramedics from another ambulance assessed the patient prior to the MSU’s arrival on the scene and considered an alternate diagnosis. An additional 10% were considered mimics after the MSU crew evaluated the patients. Only 1.5% of patients were treated in the ambulance with tPA (unpublished observations). Similar high rates of activations have been reported by MSUs from other centres [28].

These rates of stroke mimic diagnosis with MDS activation are very high and pose a high burden on health care resources. This is likely related to the paucity of information available to the MDS in their initial assessment of the patient. There is an opportunity to improve diagnostic accuracy and thus limit false ambulance activations. Education of the community on recognition of stroke symptoms may be helpful. In a recent study from Taiwan directed at increasing community awareness of stroke symptoms, approximately half the callers to the dispatchers suspected that the symptoms being experienced were related to a stroke. The medical dispatchers were more likely to activate stroke protocols and there was a higher likelihood of thrombolysis during the admission [29]. Another recent study identified that focussing on recognizing FAST symptoms, speech disturbances and a fall at onset improved the identification of stroke by dispatchers [30]. An important initiative was recently developed by the American Heart Association to improve dispatch recognition of acute stroke. Known as the “The Mission: Lifeline” stroke algorithm, it may also be very helpful with improving the appropriate recognition of the appropriate patient [31].

#### Strategies to improve diagnosis

Analysis of the content of MPDS reveals that there is too much emphasis on language and altered LOC and not sufficient importance on motor function when diagnosing an acute stroke. As language dysfunction occurs in less than 50% of patients and motor symptoms are present in more than 80% of patients, this will lead to missing stroke patients [32]. Highlighting the motor symptoms when questioning the patient may be more useful. In addition, improvement in public awareness of the symptoms of stroke will also be very helpful. Dispatchers are more likely to think that the patient is having a stroke if the callers believe that the symptoms may be related to a stroke [29].

How can we improve the specificity and not lose the sensitivity when the MDS is activated? Whereas public awareness of symptoms of acute stroke is valuable for MDS recognition, this requires constant reinforcement to allow for the heightened knowledge skills to remain intact. A review of the MPDS algorithms used for the diagnosis of acute stroke may be important. An increased emphasis on questions related to motor function should be evaluated prospectively to determine if this improves diagnosis. Such changes can be implemented with very little effort. Artificial intelligence and voice recognition are emerging technologies that hold promise and require evaluation in these settings [33,34].

### Pre-hospital EMS evaluation of stroke mimics

Recognition of an acute stroke by ambulance paramedics remains unsatisfactory with far too many mimics transferred unnecessarily to stroke centres. In a recent study from our centre, 950 patients with suspected stroke admission were transferred *via* ambulance during a single year. Following consultation by the stroke service, 42.6% of the patients were diagnosed as stroke mimics. Mimics were subdivided into neurological (55.1%) and non-neurological (44.9%) aetiologies. Seizures (19.7%), migraines (18.8%) and peripheral neuropathies (11.2%) were the most frequent neurological mimics. Cardiovascular (15.9%), psychiatric (11.9%) and infections (8.9%) were the most frequent non-neurological mimics. The ambulance paramedics had specified stroke as the primary diagnosis in half of the stroke mimic patients. In the remainder, stroke was flagged as a “suspected diagnosis”, with an alternate primary diagnosis. The “stroke code” protocol was still activated for all cases [21]. In a recent review and meta-analysis that summarized the results from seven pre-hospital studies with 6870 suspected stroke patients, 26% were diagnosed as stroke mimics. Seizures, ill-defined symptoms and ear conditions were the most frequent final diagnosis [4]. Stroke mimics were the diagnosis in 31% of 1081 in a recent study from Sweden. Epilepsy, infections, brain tumours and sequelae of prior stroke were the most common final diagnosis [35]. The presence of physicians in the ambulance seems to marginally improve the rates of diagnosis of ischaemic stroke. In a study from Poland with 805 patients referred to a neurological hospital, the rates of diagnosis of mimics were 35% for an ambulance with no physicians and 22% with ambulances with physicians [36]. Activation of “STARS” air transport *via* helicopter also experienced high rates of stroke mimics being transferred to the stroke centres. The rates of mimics were 32% of 3376 patients transferred to a single centre in a recent study [37].

Several prehospital stroke scales have been developed to improve the diagnosis of acute stroke and are increasingly being utilized by paramedics. In a review of eight prospective studies published in 2014, several clinical prehospital scores including Cincinnati Pre-Hospital Stroke Scale (CPSS), Los Angeles Pre-Hospital Stroke Screen (LAPSS), Melbourne Ambulance Stroke Screen (MASS), Medick Prehospital Assessment for Code Stroke (Med PACS), Ontario Prehospital Stroke Screening Tool (OPSS), Recognition of Stroke in the Emergency Room (ROSIER) and Face Arm Speech Test (FAST) were evaluated. Unfortunately, all prehospital stroke scales varied in their accuracy and missed approximately 30% of acute strokes. Inconsistencies in performance were likely due to sample size disparity and variability in stroke scale training. Although the point estimates for LAPSS accuracy were better than CPSS, they had overlapping confidence intervals on the symmetric summary receiver operating characteristic curve. OPSS performed similar to LAPSS whereas MASS, Med PACS, ROSIER and FAST had less favourable overall operating characteristics [38]. In a more recent systemic review of 25 studies published between 2015 and 2018, the presence of cortical signs (gaze deviation and aphasia and neglect) was most helpful in the diagnosis of large vessel occlusion (LVO). The range of sensitivity (23–99%) and specificity (24–97%) for LVO, unfortunately, remains wide and require improvements [39]. In a study from our centre, the pre-hospital blood pressure was significantly higher in acute stroke patients compared to mimics [40]. The Cochrane Database of systemic reviews considered most prehospital scales lacking sensitivity and found the Cincinnati prehospital stroke scale (CPSS) to be the most sensitive [41]. Finally, recently in a systemic review of 36 studies, the American Heart Association reported that the available stroke scales have a probability of diagnosis of LVO in 50–60% [42]. These reports show that the ability of diagnosing an acute stroke by paramedics remains low.

The introduction of portable CT scanners in the ambulance is an important recent innovation to improve early acute stroke management. The Mobile Stroke Units (MSUs) were initially introduced in Germany and the USA and are now available for the treatment of acute stroke patients in at least 30 stroke centres around the world [43]. In most instances, the MSU arrives at the site and the patient is examined by the stroke consultant or stroke fellow at the scene or *via* telestroke. Brain CT imaging is completed immediately in the MSU and where appropriate, thrombolysis initiated as the patient is transferred to the hospital. MSUs reduce the time from stroke alarm to treatment by 25–40 min and increase the rate of intravenous tPA use without an increase in haemorrhage risk. MSU also improve the prehospital accuracy of stroke diagnosis and triage. One recent randomized study showed that with an MSU the triage accuracy of acute stroke including mimics was 100% with an MSU compared to 70% with standard LAMS-based prehospital triage [44]. In a recent study evaluating mimics in MSU, 423 patients were included for analysis. The stroke mimics (29.3% of patients) were easily recognized by the MSU team and included seizures, infections and migraine as the most common diagnosis [45]. Only 1.6% of mimics received thrombolysis compared to 34.5% of patients with acute stroke. A telestroke mimic score (TM-score) was used to identity suspected mimics [3]. The introduction of CTA and CTP in the MSUs improves the diagnosis of LVOs in the field **[**28]. An important advantage of the MSU is that with the expert evaluation and imaging, the patient can be sent back to their local hospital in rural settings if they are mimics [46]. MSUs are expensive and are available in a few selected stroke programmes. The likelihood for wider availability of the technology is low. Other methods that will lead to better diagnosis and an increase in the rates of thrombolysis are urgently needed.

Given the modest ability to diagnose acute stroke with current stroke scales, there have been recent attempts to introduce novel technologies in the prehospital diagnosis of acute stroke. The technologies being tested fall into three main categories; blood biomarkers, portable imaging and video linking between the ambulance and consultants. The number of studies is small with blood biomarkers and to date have not shown much promise [47]. Imaging devices are testing the usefulness of infra-red screening for ICH and EEG for detection of large strokes. Available data with infra-red shows a lack of specificity in accurately differentiating an acute ischaemic stroke from an ICH. There is also minimal experience with diagnosing stroke mimics with these devices [47]. None of the dry-EEG studies have reported results on their experience with evaluation of acute stroke in the ambulance. Finally, 15 observational and controlled studies have utilized video transmission between ambulance and hospital ED. The preliminary evidence is encouraging and the detection of stroke mimics appears to be comparable to the incidence in the ED. There is also evidence that the use of such technology improves the door-to-needle times for thrombolysis [48]. Other devices that are undergoing testing for improved detection of stroke include accelerometers, microwaves, radiofrequency, transcranial Doppler ultrasound and volumetric impedance phase shift spectroscopy. All these technologies are in very preliminary stages of development [47].

#### Strategies to improve diagnosis

A large number of prehospital stroke assessment scales for diagnosis of LVOs with most showing diagnostic accuracy of less than 50% indicates that improvements are necessary for accurate diagnosis of acute stroke [42].

Can we improve the recognition of stroke mimics by the paramedics? Educational interventions aimed at improved recognition may improve stroke diagnosis in the ambulance [23]. Two recent studies evaluating two separate diagnostic methodologies hold promise. A study from Madrid utilizing an improved assessment tool (M-DIRECT), included the patient’s age, blood pressure, gaze preference and motor weakness, achieved a sensitivity of 76% and a specificity of 82% in diagnosis of LVOs [49]. Another study from Stockholm evaluated all code-stroke patients within the city for one year. Patients with moderate-severe hemiplegia were reviewed during transfer *via* a telephone consultation with physicians (11% of patients). The positive predictive value was 41% with a very impressive negative predictive value of 91% **[**50]. The introduction of video-conferencing with the physician also appears to hold promise [51**]**. These advances will be particularly important to help in patients with suspected LVOs in whom bypass protocols have been shown to improve outcomes with thrombectomy [52]. The introduction of MSU remains the best means for improved diagnosis of acute stroke and reduced transfer of stroke mimics to the stroke centre. Our experience with the MSU shows that it is the combination of the stroke expertise and the portable CT scanner that is very useful in ruling out some mimics while the patient is being evaluated in the ambulance [53]. MSUs are however expensive and unlikely to be widely available in stroke management. Utilizing videoconferencing between the paramedics and the stroke consultant in selected high-risk patients may be a potentially more cost-effective compromise to lower transfer of stroke mimics to stroke centres **[**50]. Newer technologies that allow for direct tele/video interaction being the ambulance and the stroke team will also limit the transfer of stroke mimics to stroke-ready hospitals.

### Emergency department evaluation for stroke mimics

Patients with suspected acute stroke are rapidly evaluated in the ED if they are within the thrombolysis window with the intention to reducing the “door-to-needle” times [54]. There is a correlation between shortening “door-to-needle” times and an increase in the risk of treating mimics with tPA [55]. Within minutes of arrival in the ED, the patient is in the CT scanner for a non-contrast head CT scan. In eligible patients (up to 4.5 h from onset of symptoms), treatment with tPA is often initiated in the CT bay before further imaging (CTA/CTP) is completed. This ultra-fast assessment and treatment, however, comes with a cost. There is a serious risk of treating a stroke mimic as the evaluation may not be sufficiently detailed and the limited imaging may not detect non-vascular aetiologies.

In the ED, stroke mimics are diagnosed under two common scenarios; emergent evaluation for reperfusion treatment and second, in the assessment of stroke patients beyond the thrombolysis window to ascertain the diagnosis of ischaemic stroke. Patients presenting beyond the 4.5-h time window undergo a more detailed multi-modal cranial CT assessment for possible reperfusion intervention and are therefore less likely to diagnose a mimic as an acute stroke. Stroke mimics can expeditiously be diagnosed in busy CSC affiliated EDs with rapid access to vascular neurologists and multimodal imaging, especially when MRI is available to screen patients with acute stroke. In a recent study, 1361 patients presenting within 4.5 h with sudden onset symptoms suspected of being a stroke were imaged with MRI. Mimics were diagnosed in 38% of patients admitted as acute stroke. Migraine, functional (conversion disorder), seizures and vertigo were the most common mimic diagnosis [56]. MRI is however not widely available as a screening investigation in EDs.

Functional disorders can sometimes be particularly difficult to diagnose when there are significant time constraints in decision making for thrombolysis. The clinical bedside evaluation remains the best method available to distinguish between an acute stroke and a functional mimic. A careful examination by an experienced neurologist is often required to note the signs suggestive of a functional disorder. These include the demonstration of a Hoover’s sign (hip flexion and extension testing reveal inconsistency in attended *vs.* unattended movement in affected leg), “give-away” weakness and downwards drift without pronation as fairly characteristic for functional weakness. Facial weakness may be associated with ipsilateral lip-pulling and orbicularis oculi contraction (see Table 2) [57]. The absence of imaging abnormalities, especially with multimodal CT scanning or MRI can be very helpful in the diagnosis. Munchhausen syndrome may be considered in patients coming repeatedly and demanding tPA. In one recent report (10 of 335 (3%) of patients presenting with suspected acute stroke were thought to have Munchhausen Syndrome [58].

Stroke algorithms have been designed for early detection of stroke mimics in the ED [59]. In a 9-point score, the specificity for diagnosis of stroke mimic was 100% if the score was higher than 5 [59]. While such scores may be helpful, they are seldom used in clinical practice when patients are examined in ED.

Post-stroke recrudescence (PSR) is an important and underreported cause of stroke mimic [11]. PSR has been reported within weeks to years following an acute stroke or ICH. The duration of symptoms is typically brief and usually resolved within less than 24 h. Deficits are often abrupt, mild and commonly affect motor-sensory or language function. PSR should be considered in patients with a previous history of stroke who develop symptoms in the distribution of the previous injury. Prompt recognition often requires an MRI that reveals the old stroke but no new lesions responsible for the neurological worsening. The correct diagnosis will help minimize rt-PA use in this group of patients [11] Interestingly, mimics may be more frequently seen than recurrent stroke in patients with a history of previous stroke. The diagnosis of mimics may be challenging in such patients, especially when there are residual deficits from the prior stroke [60].

There is wide variance in the use of rt-PA in stroke mimics. Two large stroke thrombolysis registries published their experience recently. In the SITS international stroke thrombolysis registry with 10,436 treated subjects, only 429 (4.1%) treated patients were identified as stroke mimics. Functional disorder (30.8%), migraine (17.5%) and seizures (14.2%) were the most common mimics treated [61]. The larger “Get-With-the-Guidelines-Stroke Registry” also reported their experience with 72,582 treated patients between 2010 and 2017. Thrombolysis was given in 3.5% of stroke mimics. Migraine, functional disorder and seizures accounted for 38.2% of subjects [62]. These numbers likely underrepresent the true extent of the problem due to inherent limitations of self-reporting in voluntary registries. The rates of thrombolysis in mimics from hospital-based experiences reveal higher rates with one large series reporting 18% of non-stroke patients receiving tPA [63]. Treatment in stroke mimics adds unnecessary expense and prolongs observation in high-intensity units [63]. Patients may carry an incorrect diagnosis of ischaemic stroke and be unnecessarily treated with long-term statins and antithrombotic therapy. In patients treated with rt-PA, the risk of symptomatic ICH is however significantly lower in stroke-mimics when compared to ischaemic stroke [64].

Can imaging help limit the use of tPA in stroke mimics? Non-contrast cranial CT is very often normal following acute stroke and is generally sufficient to initiate thrombolysis if the clinical syndrome does not raise alarms of a stroke mimic. In most CSCs, multimodal CT studies including CTA and CTP are initiated following the plain CT as the tPA is being infused. The CTP/CTA is frequently abnormal and reveals the site of arterial occlusion and the area of ischaemic core/penumbra. In a recent study from France 753 patients treated with tPA were assessed with CTP/CTA. Multimodal imaging was negative in 142 (18.9%) of patients. The 24-h MRI revealed a lesion in 44.4% of patients with normal imaging. The remainder of patients in whom no lesions were discovered was likely mimics [65].

MRI is superior to CT at capturing mimics during the very early time following the acute event and is the imaging modality of choice if readily available. A pooled meta-analysis of 3236 patients revealed a prevalence of 6.8% of DWI-negative stroke [66]. While it is rare for a moderate to large size cortical stroke to have negative DWI imaging, the MRI may be normal with small posterior circulation stroke syndromes involving the brainstem. In one study with 1361 “code stroke” patients with suspected stroke, MRI was used as the initial imaging tool 24 h a day with close supervision by a stroke neurologist. In 38% of cases, imaging was able to help establish the diagnosis of stroke mimic [56].

**Figure 2.**
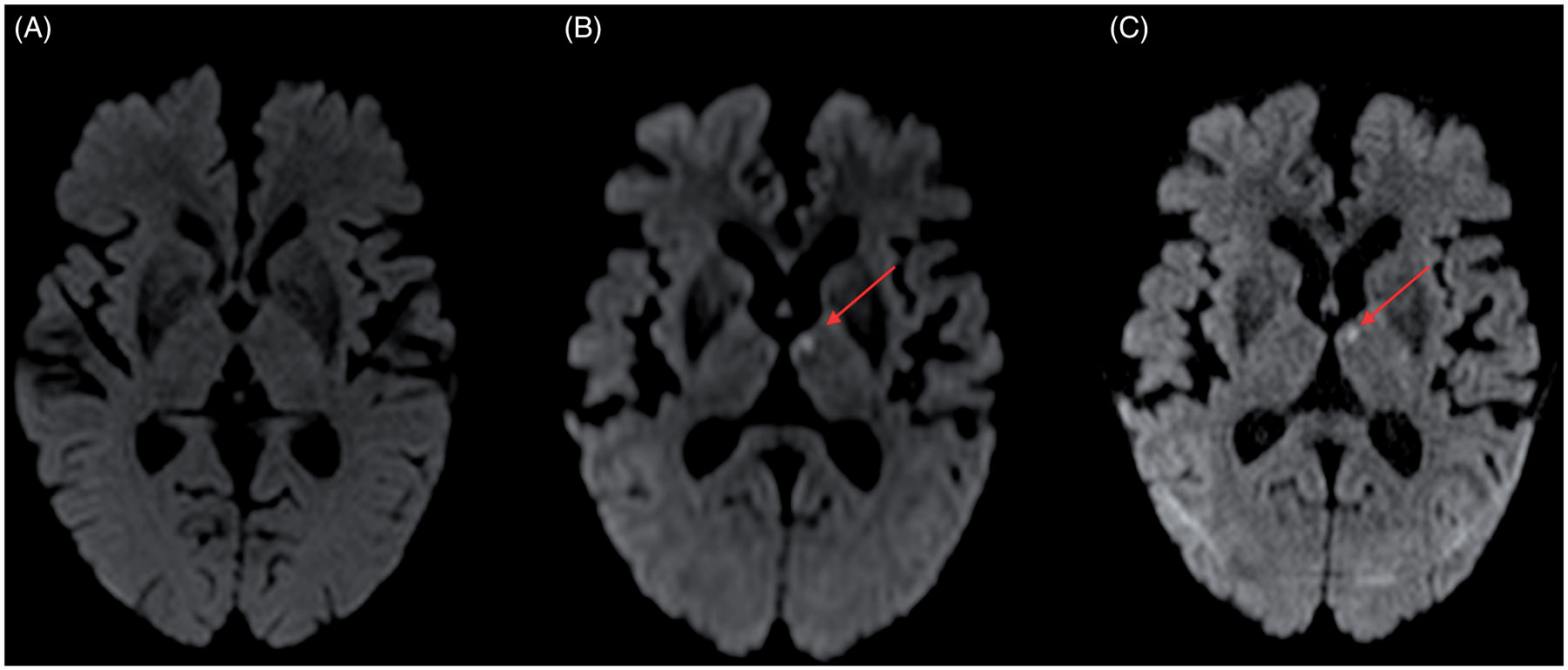
Patient presented with sudden onset right-sided numbness and no other deficits. Routine computed tomography (CT) was normal. A: Diffusion-weighted (1.5 T) magnetic resonance imaging (DWI-MRI) was normal. B,C: High resolution 3 T MRI (DTI and DWI-isotropic voxels) revealed left thalamus infarction.

The distinction between a mimic and stroke can be particularly challenging when the MRI does not reveal an acute ischaemic infarction but the neurologist strongly suspects a stroke, i.e. image negative stroke. In such situations, the distinction from a stroke mimic can be extremely challenging. In a recent survey of 65 neurologists, vascular neurologists and neurohospitalists, the agreement in 10 case-vignettes was modest when symptoms were plausible for acute stroke (aphasia and hemiplegia) and MRI did not reveal a new lesion [67]. Such uncertainties in making an accurate diagnosis of stroke or mimic are worrisome given the high frequency of stroke mimics in these situations. In patients where the suspicion of an ischaemic stroke is high and MRI is normal, high-field 3 T MRI imaging may show a lesion (see Figure 2). Methods to improve rapid diagnosis of stroke mimics are important to avoid unnecessary use of thrombolysis and utilization of acute care beds.

#### Strategies to improve diagnosis

The ED remains the most important location where recognition of stroke mimics is time-critical, especially when the patient arrives within the window for thrombolysis. The rates of thrombolysis in stroke mimics are likely higher than registry reports [61,62]. The diagnosis of “aborted stroke” is problematic. It is a term frequently used to describe the situation when imaging, especially MRI-DWI, does not reveal an ischaemic lesion in tPA-treatment patients [68]. Most such patients are likely stroke mimics, especially when the symptoms suggest anterior circulation localization [69]. Correct diagnosis is especially important because misdiagnosis carries with it the risk of the inappropriate lifetime use of stroke prevention medication, the psychosocial problems and insurance implications associated with it.

**Figure 3.**
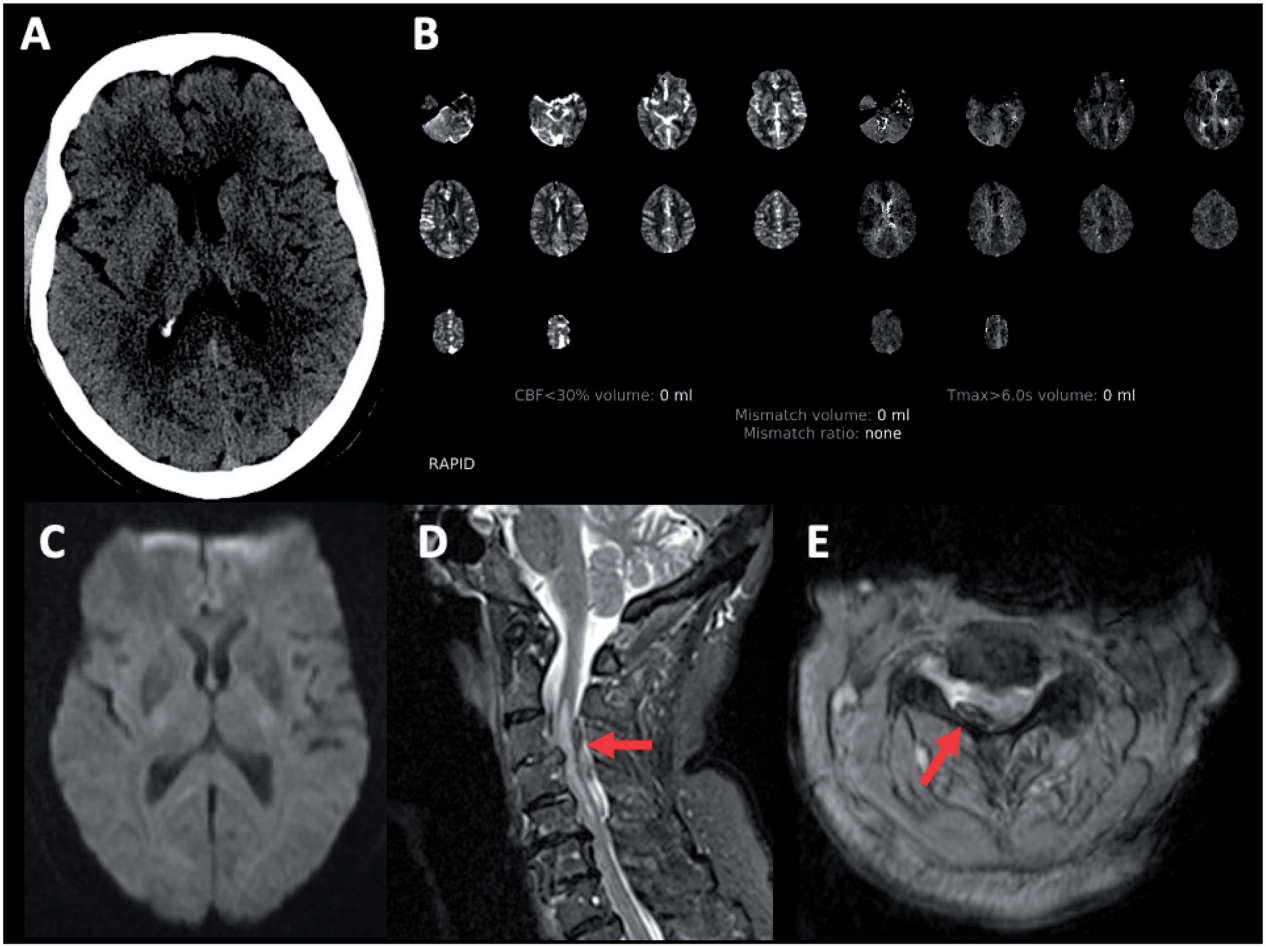
80-year-old female with atrial fibrillation on apixaban. She presented with a 90-min history of sudden onset right arm and leg weakness sparing the face with normal sensation. A,B: Axial computed tomography (CT) head, CT angiogram (not included) and CT perfusion were unremarkable. Tissue plasminogen activator (tPA) was no given because the patient is on an anticoagulant. C: Diffusion-weighted magnetic resonance imaging (DWI-MRI) was normal. D,E: Sagittal and axial MRI spine revealed right posterolateral epidural haematoma, a multifocal severe spinal canal stenosis, and spinal cord oedema.

Misdiagnosis and inadvertent treatment with tPA in stroke mimics can also sometimes be dangerous and lead to serious injury or death. Aortic arch dissection can present with focal neurological symptoms from occlusion of the carotid or vertebral artery. In a recent large series of 2874 code strokes, 15 patients (0.5%) had an aortic dissection. A decrease in level of consciousness, pain and low blood pressure were associated with abnormalities that were helpful in making the correct diagnosis [70]. Similarly, trauma can result in epidural (see Figure 3), subdural and brain haematoma that can at times be difficult to diagnose even with careful imaging. If the history of trauma is not evident, especially in patients with cognitive dysfunction, speech abnormalities or hemiplegia, the patient may be treated with tPA with serious complications [71].

How can we improve our ability to diagnose a stroke mimic in the ED? The quick access to stroke expertise and proximity of imaging facilities to ED can be helpful if available. The clinical examination remains the most important tool for the diagnosis of a functional disorder (Table 2). The combination of a quick clinical examination and multimodal CT scanning is sufficient for diagnosis in most instances. Patients with cortical vascular syndromes on examination (e.g. weakness, sensory neglect, visual field deficit and gaze preference) will often have large vessel intracranial occlusion when examined with CTA [16]. Such patients seldom pose diagnostic challenges. In patients with partial syndromes, volume and/or blood flow deficits evident on CTP may be helpful, especially when the distal intracranial occlusion may not be visible on the CTA. The presence of normal CT, CTA and CTP should alert the physician to the possibility of a stroke mimic. CTP may however frequently show non-specific findings in mimics. These are however discordant with the clinical examination [72]. CTP can, however, be unremarkable in patients with small lacunar sub-cortical stroke and therefore be interpreted in the context of the clinical examination. Such lesions can become apparent with MRI, especially when high field imaging is used (see also Figure 2). While small infarctions, especially in the brainstem, may be missed on routine MRI, the absence of a lesion on MRI should raise the possibility of a stroke mimic, especially in the presence of poorly localizing symptoms. It is safer to wait and seek expert opinion and not rush to thrombolysis in patients with unclear symptoms and normal imaging.

### Telestroke evaluation

Telestroke allows the neurologist to evaluate suspected acute stroke patients at distant centres that have limited stroke expertise [73]. Patients are examined remotely and the brain CT images are available for review as they simultaneously become available. Because of ease of use and greater availability and cost-effectiveness, an increasing number of acute stroke patients are being managed *via* telestroke. In these circumstances, as the patient is not physically examined by the neurologist, the likelihood of treating a stroke mimic is high. The likelihood of stroke mimics treated with tPA was evaluated in a study of 829 telestroke managed patients [74,75]. The frequency of stroke mimics was 23%. Stroke mimics were younger, often had a previous history of migraines or seizures and had fewer vascular risk factors [3]. The investigators subsequently created a TM-score and validated it in a study of three large telestroke programmes in the USA and Germany. Overall there were 32.6% stroke mimics in the multicentre study (mimics ranging of 24–41% between centres) [3]. The variance between centres was attributed to the experience of the telestroke neurologists in each of the three sites. The lower frequency of mimics was noted from the single centre in Germany with the most experience with telestroke [3]. In another study from South Carolina, the rates of stroke mimic treatment were compared between in-hospital and telestroke patients. Significantly, higher rates of mimics were thrombolysed in the telestroke group (27.2 *vs.* 14.8%). The rates of negative imaging were also higher in the telestroke group (40 *vs.* 34.1%). The rates of ICH and good outcomes at 90 d as measured on the modified Rankin Scale were similar between the two groups [75]. Finally, in a report from Washington Hospital Comprehensive Stroke telestroke programme, the discrepancy between treating stroke mimics was higher when MRI imaging was compared to CT imaging in unclear cases. Compared to 0.5% mimics thrombolysed at the comprehensive centre where MRI was the imaging of choice, 16% stroke mimics were treated at telestroke sites where only CT was available for brain imaging [76].

#### Strategies to improve diagnosis

The increasing use of telemedicine while safe remains a challenge for stroke mimic recognition [77] and inadvertent use of thrombolysis is substantially higher in such situations [76]. Particular care must be taken in the distant examination and the liberal use of CTA/CTP to ensure that thrombolysis is avoided in stroke mimics, especially when symptoms are mild or atypical. This remains an area of particular concern and requires prospective studies to improve the rates of errors, especially with newer programmes. A prospective registry monitoring the rates of treatments in mimics will be very useful.

## Common conditions that present as stroke mimics

There is a wide list of conditions that can present with stroke-like symptoms and be mistaken for acute ischaemic infarction. The list below is not exclusive but a brief discussion of common conditions that may be mistaken for an acute stroke. Recrudescence, functional disorders, migraine aura and seizures are amongst the most likely diagnosis that requires recognition to improve our rates of inappropriate thrombolysis.

### Recrudescence

#### Presentation

Recrudescence refers to the re-emergence of the previous stroke-related deficits in the settings of metabolic, infectious and toxic dysfunction. The diagnosis requires an MRI which shows the old stroke and reveals no new DWI-MRI deficits. Symptoms are mostly short-lived and show improvement within 24 h in most subjects. Recrudescence can develop within weeks to years after the previous stroke [11]. The recrudescence under metabolic, infection, fatigue or sedative medications is likely due to functional suppression of compensatory cerebral networks [78].

#### Management

Correct diagnosis is important as the condition is under-recognized. Management revolves around treatment of the associated medical condition.

### Functional

#### Presentation

Presentation is very varied and requires a high degree of suspicion. In most large series, it is the most frequently thrombolysed stroke mimic. Previous psychiatric history common, symptoms atypical and fluctuating. Physical examination shows inconsistent findings with repeated examinations (see Table 2). Imaging is normal or reveals no DWI-MRI lesions [79**].** The prognosis for full recovery is unfortunately not very good with more than one-third of patients reporting same or worse deficits during follow up [9].

#### Management

Reassurance that there is no structural lesion to explain symptoms and psychiatry consultation is recommended.

### Migraine

#### Presentation

Migraine aura without headache is a frequent stroke mimic Symptoms usually “positive”, including paraesthesia and visual phenomenon, gradual in onset with “slow march” through different vascular territories. Basilar migraine uncommon and can present with vertigo, dysarthria, ataxia and decrease LOC. When symptoms are prolonged, suspect hemiplegic migraine. Imaging, including DWI-MRI, is normal [80].

#### Management

Recognition of diagnosis, especially hemiplegic migraine important as symptoms recurs frequently. Treat headache symptomatically and consider prophylaxis if recurrences frequent. CTP imaging is normal and can help differentiate from acute stroke [81].

### Seizures

#### Presentation

**Figure 4.**
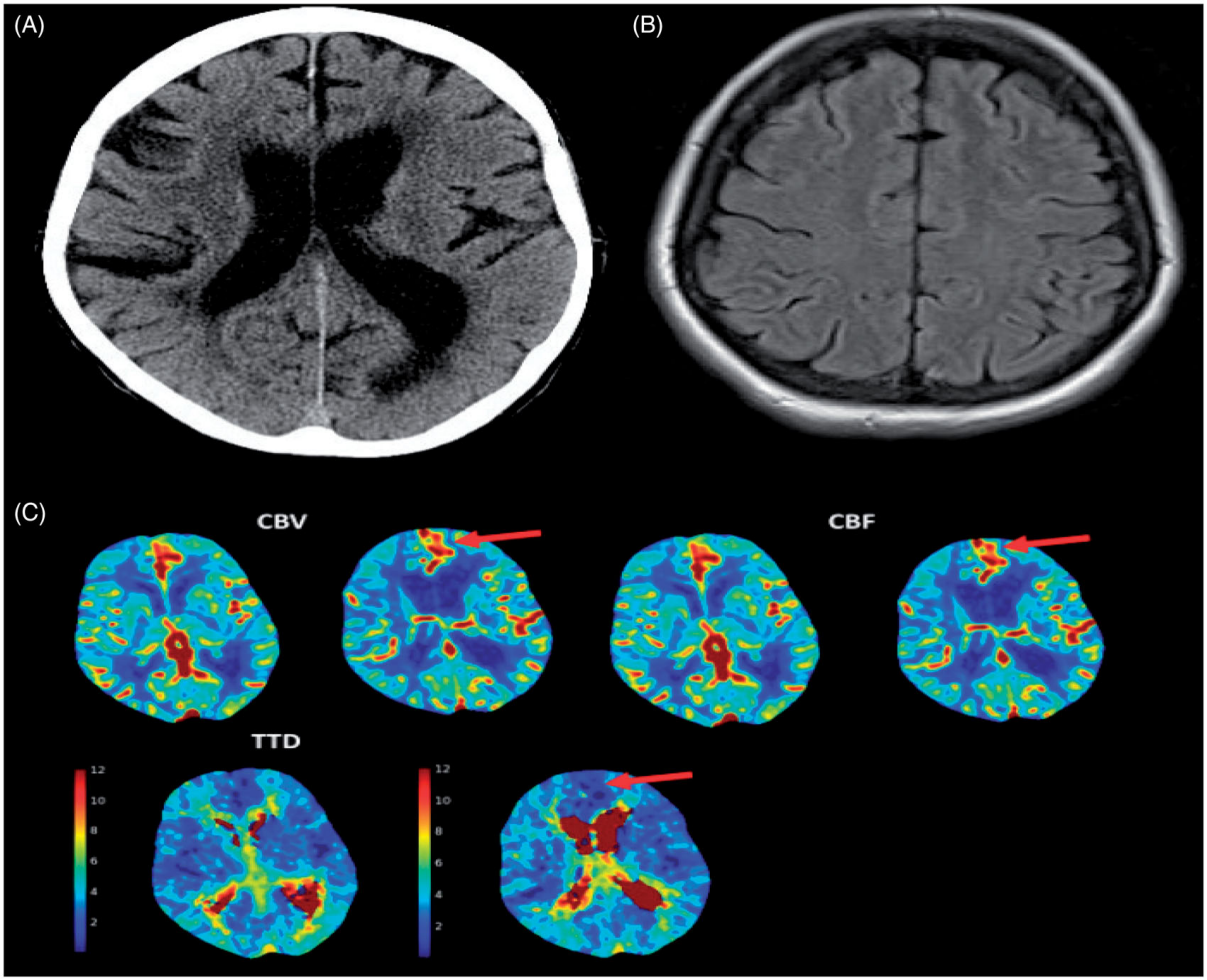
An 85-year-old female presented with expressive aphasia. A,B: Axial computed tomography (CT) head, CT angiogram (not included), magnetic resonance imaging (MRI) of the brain that also included diffusion-weighted imaging were unremarkable. The patient was discharged with the diagnosis of transient ischaemic attack (TIA). After discharge, the patient continued to have recurrent similar episodes. C: CT perfusion during one of her episodes demonstrated left frontal focal hyperperfusion characterized by increased cerebral blood flow (CBF), cerebral blood volume (CBV) and decreased time to drain (TTD). The patient was diagnosed with focal seizure with preserved awareness.

Focal seizures are common and may be seen in patients with a previous stroke [82]. Post-ictal Todd’s and muteness paralysis may be confused for acute stroke especially if the seizure was unwitnessed. Altered level of consciousness is common. Generalized seizures may also be an initial manifestation of an acute stroke. Recovery is rapid in most patients although rarely non-convulsive status epilepticus may present with prolonged aphasia or motor weakness (see Figure 4).

#### Management

Active seizure is a medical emergency and requires immediate intravenous medications to stabilize the patient. Long-term management will require a neurology consultation. Risk of recurrence high.

### Peripheral vertigo and dizziness (inner ear dysfunction)

#### Presentation

A common presentation that is often difficult to differentiate between peripheral (e.g. inner ear) or central mechanism (e.g. cerebellar or brainstem stroke). The presence of additional brainstem signs (dysarthria, diplopia, ataxia, weakness and numbness) may be helpful but not always present. Isolated vertigo or unsteadiness may be the only symptom of a cerebellar stroke. A DWI-MRI may be important for diagnosis. MRI can also be normal with small brainstem lesions.

#### Management

Peripheral vertigo can be very disabling but responds well to rest and supportive care. Important to repeat imaging as early DWI-MRI may be falsely normal. May require ENT consultation if symptoms recurrent and Meniere’s disease suspected.

### Multiple sclerosis (MS)

#### Presentation

Acute MS presentation can occasionally be mistaken for acute stroke. Younger age, prior history and multiple abnormalities on neurological assessment may be helpful. The diagnosis is particularly difficult if it is the first attack of MS. While CT may be normal, MRI will show white matter lesions in typical locations and often additional asymptomatic lesions [20]. It is important to remember that the event may be an acute stroke in a patient with MS. The risk of stroke is increased by 28% in MS [83].

#### Management

The diagnosis is rarely entertained until after the completion of the MRI. Once the diagnosis is confirmed the patient will require specialized care by an MS expert.

### Neoplasia and other space-occupying lesions

#### Presentation

The clinical presentation poses no problems with intracranial mass lesions. Occasionally, low-grade gliomas can present with focal seizures and post-ictal Todd’s paralysis. The brain tumour on imaging may be confused for an ischaemic stroke. Typically, the lesion on CT and MRI do not correspond to vascular distribution and may have surrounding oedema.

#### Management

Treatment depends on the underlying condition. Seizures may require anticonvulsants.

## Conclusions and recommendation

Misdiagnosis of acute stroke is a major health care problem. When up to 40% of patients admitted to the hospital with suspected acute stroke have an alternate diagnosis, there are important implications including the risks associated with emergent administering the incorrect treatment and the inappropriate long-term stroke prevention treatment when it is not required. In addition, the unnecessary utilization of the trauma beds, extensive hyper-acute investigations and thrombolysis carries a significant unnecessary front-end cost.

Mistaking a mimic for acute stroke occurs at all levels of contact between the patient and the health care. Although it is frequent with medical dispatchers, mimics do not pose serious issues at this level. In most instances, sending an ambulance is not only important for a suspected stroke but for most conditions that mimic a stroke. However, if the paramedics make an incorrect diagnosis this has consequences. The danger with the unnecessary transport of stroke mimics to a stroke-designate hospital is that it can lead to inefficient utilization of limited resources along with the inherent risks in delaying treatment where it may be urgently needed. Traditionally, the ambulance services have not been very good at separating mimics from acute ischaemic stroke. While the introduction of some new stroke assessment scales has improved the recognition, it has unfortunately not resulted in any meaningful increase in thrombolysis or time to treatment [49]. The MSU is definitely an improvement but very expensive and very unlikely to be widely available for stroke care. The introduction of telephone or video-link with the stroke physician seems promising but requires further studies [50].

Careful physician evaluation in the ED will improve rates of correct diagnosis of acute stroke. In considering the possibility of stroke mimics, it is useful to remember that acute stroke presents in well-defined syndromes. Typical symptoms, when combined with characteristic CT abnormalities, including cortical effacement, loss of insular ribbon and haziness of the caudate, are the hallmark of early ischaemic stroke presentation. The presence of intracranial arterial occlusion and mismatch on CTP, in appropriate clinical settings, is sufficient to make the diagnosis of acute stroke. Very early, CT imaging may be normal in stroke mimics but over time reveals the typical patterns of the underlying disease (for example brain tumour or infection). MRI is usually very helpful, especially when conversion disorder is suspected. MRI may also show no new lesions in patients with migraine, seizures or recrudescence related to an old stroke.

Clinical clues, screening blood work-up and brain imaging can detect most commonly seen stroke mimics (Figure 1; Table 2). The symptoms are gradual in onset, not anatomically distinct and classically fluctuate and improve as the underlying condition is treated. The clinical examination can, however, be challenging in patients with functional disorders. If not recognized in time, such patients frequently get thrombolysis. While the weakness, sensory changes and speech dysfunction can sometimes be difficult to differentiate, gaze deviation is uncommon in patients with functional disorders. Despite the significant focal deficits, the patient seems to be aware of their surroundings and tends to follow the examination with their eyes. Similarly, arm drip without pronation of the fingers is very suggestive of functional weakness (see Table 2).

It is also important to remember that non-specific neurological symptoms can also occasionally be associated with acute stroke. In one recent series, 13.5% of patients with “low-risk” symptoms (for example; dizziness and numbness) were found to have DWI-positive lesions. The risk of recurrent ischaemic events was significantly higher in DWI-MRI patients, especially if the lesions were multiple [17]. Missing the correct diagnosis of acute ischaemic stroke or TIA has consequences. The risk of recurrent ischaemic events is high in the initial days following the onset of symptoms. As secondary preventive measures can reduce the risk of vascular events, accurate early diagnosis is important [84].

Missing a stroke mimic in the ED has important adverse consequences in two situations. First, in patients presenting very early to the hospital, there is the risk of thrombolysis in a stroke mimic. In our experience, this risk is highest in patients with functional disorders, migraine aura, focal seizure and peripheral vertigo. Brain imaging is normal or non-diagnostic in such disorders and diagnosis depends on careful bedside assessment (see Table 2). Thrombolysis can cause severe harm in other rarer stroke mimics including, aortic dissection, bacterial endocarditis, epidural/subdural haemorrhage and trauma (see Table 1). Appropriate imaging in these conditions is often helpful in making the correct diagnosis. Second, not recognizing the correct diagnosis will delay treatment of the disease that is manifesting as the stroke mimic (e.g. appropriate treatment of focal seizures or conversion disorder). As mentioned above, there is the added risk associated with long-term unnecessary use of stroke prevention medications.

The treatment of stroke-mimics depends on the underlying condition as outline above with common conditions that may be mistaken for an acute ischaemic stroke. In patients where tPA is administered inadvertently, it should be stopped as soon as the diagnosis becomes apparent. If the drug has been infused, the patient will still require close observation for 24 h according to protocol. It is important that the diagnosis is correctly recorded, especially in patients with conversion disorder where there is a high likelihood of repeated hospital visits. Such patients ideally require a psychiatric assessment. Recurrence is also likely with migraine, focal seizures and in patients with a pre-existing stroke (recrudescence). Treatment of the underlying conditions is important to decrease the risk of recurrence.
